# Associations between Maternal Nutrition and the Concentrations of Human Milk Oligosaccharides in a Cohort of Healthy Australian Lactating Women

**DOI:** 10.3390/nu15092093

**Published:** 2023-04-26

**Authors:** Caren Biddulph, Mark Holmes, Trong D. Tran, Anna Kuballa, Peter S. W. Davies, Pieter Koorts, Judith Maher

**Affiliations:** 1 Centre for Bioinnovation, University of the Sunshine Coast, Maroochydore DC, QLS 4558, Australia; 2 School of Health, University of the Sunshine Coast, Maroochydore DC, QLS 4558, Australia; 3 School of Science, Technology and Engineering, University of the Sunshine Coast, Maroochydore DC, QLS 4558, Australia; 4 Child Health Research Centre, University of Queensland (UQ), St. Lucia, QLS 4072, Australia; 5 Department of Neonatology, Royal Brisbane and Women’s Hospital, Herston, QLS 4029, Australia

**Keywords:** lactation, maternal diet, body composition, human milk oligosaccharide, breastfeeding

## Abstract

Human milk oligosaccharides (HMOs) are complex glycans associated with positive infant health outcomes. The concentrations of HMOs in the milk of lactating women are associated with substantial intra- and inter-individual differences and may be influenced by maternal physiological and/or nutrition-related factors. The primary aim of this study was to explore potential influences of short-term maternal diet and current body composition on HMO profiles in mature human milk. Milk samples were collected at 3–4 months postpartum from 101 healthy Australian women using standardised procedures, and analysed for macronutrients (lactose, fat, and protein). In addition, HMO concentrations were analysed using liquid-chromatography mass-spectrometry (LC-MS). Maternal dietary data were collected using three validated 24-h dietary recalls, and the body composition of a subgroup of mothers was assessed by DEXA scans (*n* = 30). Most (79%) of the women were secretor-positive. Individual nutrients were not significantly correlated with HMO concentrations after correction for multiple comparisons (*p* > 0.05), except for dietary folate intake. DEXA scans revealed no associations between HMO profiles and maternal body composition during established lactation. The study findings suggest a lack of clear and consistent associations between maternal nutrition and HMO concentrations in mature human milk from healthy lactating women with adequate dietary intake. The prevailing influence of genetic variation in lactating mothers may overshadow any impact of maternal nutritional and/or physiological status on HMO composition in mature human milk.

## 1. Introduction

Human milk is a unique and ideal source of nutrition for most infants and is comprised of essential nutrients and bioactive factors that influence their growth, health, and development [1]. Human milk oligosaccharides (HMOs) are a group of complex, bioactive glycans that are abundant in human milk and have known health-promoting benefits [2]. They function as prebiotics to support the growth of beneficial gut bacteria, modulate the infant’s immune system, act as soluble receptor glycoconjugates, and provide infants with a source of sialic acid for brain development [3,4]. Research on HMOs continues to proliferate, though much work is centered on the physiological and immunological properties of certain HMOs. Questions remain regarding the variability in HMO profiles amongst and between women, and the role of maternal factors that potentially drive these differences. The variation in HMO composition is primarily due to maternal genetic factors, with numerous studies reporting on the effects of polymorphisms of the secretor and Lewis genes on the amount and diversity of HMOs [5,6,7]. Lactation stage, environmental factors such as geographic location and seasonality, and maternal influences such as nutritional status may also affect HMO composition [8,9,10,11]. However, a recent scoping review highlighted the disparities in the limited information on the effects of maternal dietary intake during lactation and maternal postpartum body composition on HMO profiles [12].

Dietary intake as an exposure remains problematic to measure accurately in lactating women, due in part to the complexity and number of dietary elements that must be accounted for. Variability in the approach to assessing dietary intake and the choice of dietary assessment tools also confound comparisons across studies. Some studies have assessed dietary intake during pregnancy (as opposed to during lactation) [8,13], and those that have assessed postpartum diets had a small study size (*n* = 8–14) [14,15]. Two larger, more recent, studies in China used single-food-frequency questionnaires in the postpartum period to assess usual maternal diet, and analysed a limited number of HMOs [16], or only sialic acid levels [17]. To our knowledge, and at the time of undertaking this study, there were no reports of investigations into short-term maternal dietary intake (i.e., in the 24 h preceding human milk sample collection) in free-living individuals, and none in Australia. Another key finding from the above-mentioned scoping review was that there may be some evidence for associations between maternal body composition and HMO profiles [7,9,10,18,19,20]. The validity and appropriateness of BMI as a measure of body composition during lactation, as reported in previous work, is questionable, as it is a proxy measure of body size, not body composition. No studies to date have used gold-standard body-composition-assessment tools, such as Dual-Energy X-ray Absorptiometry (DEXA) scans [12].

The aim of this research study was to investigate the associations between maternal nutrition and HMO concentrations, using validated nutrition assessment tools. The first objective was to explore the associations between short-term maternal dietary intake during lactation and the composition of HMOs in mature human milk samples from healthy Australian women (*n* = 101). The second objective was to investigate associations between maternal postpartum body composition and HMOs in mature human milk samples from a subgroup of Australian women (*n* = 30), using DEXA scanning techniques.

## 2. Materials and Methods

**Figure 1:**
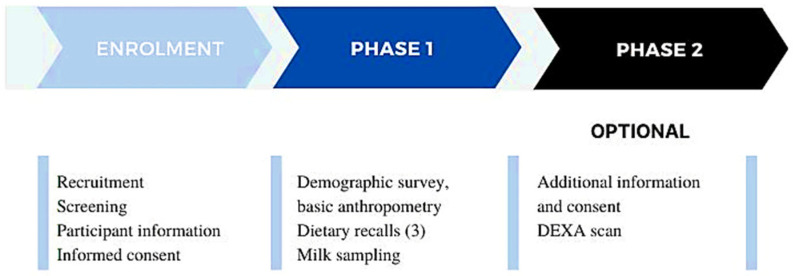
Study design depicting two phases of investigation and the flow of participants through the study.

This observational study featured a cross-sectional design with convenience sampling of a group of free-living, community-based, lactating women in Australia. The study consisted of two phases to investigate both short-term dietary intake and longer-term nutritional status using validated nutrition assessment tools. Phase 1 encompassed online surveys (one demographic questionnaire and three 24 h dietary recalls), followed by a standardised milk-sampling protocol. Phase 2 involved body composition assessments using dual X-ray absorptiometry (DEXA) technology. This combination of investigations exemplifies robust dietetic nutritional investigation and is illustrated in Figure 1.

Mothers were eligible if they were breastfeeding term singletons with an established milk supply at 3–4 months postpartum and were in general good health.

### 2.1. Maternal Investigations

The first survey completed by participants captured relevant exposure variables relating to demographics, breastfeeding and birth information, and selected health and nutritional data, as reflected in Table 1. Data points were informed by existing surveys [8,9] to allow for comparison across data sets where possible. Maternal pre- and postpartum BMI were classified using the standard equation Weight/Height^2^ (Quetelet’s index; kg/m^2^) [21]. Gestational weight gain and patterns of weight gain/loss over pregnancy and the lactation period were extrapolated and compared.

Maternal dietary intake during lactation (i.e., in the short-term period around the time of milk sampling) was assessed using the Australian version of the Automated Self-Administered 24-Hour Dietary Recall (ASA24^®^). The tool has been previously validated in pregnant women [22] and in women of childbearing age [23]. Participants completed three self-administrated 24 h dietary records, noting each food, fluid, and dietary supplement consumed. No dietary recommendations were given to the participants before the study. Images assisted with portion size estimation, and food codes from the Australian Food Supplement and Nutrient Database (AUSNUT) 2011–2013, were automatically assigned to over 30 nutrients. Mean values from three days of recalls (two weekdays and one weekend day) were used to estimate usual dietary intake, and the final recall of items consumed the day directly prior to milk sampling was used as “24-h dietary intake”. An accredited dietitian checked all survey responses for completion and for potential under/over-reporting of dietary energy [24]. An additional secondary analysis was performed to investigate certain prebiotic foods in the mothers’ diets, after collating a database from selected publications [25,26,27].

Maternal body composition was assessed using a validated four-compartment model, DEXA, and was offered to all participants as an additional, optional part of the study. Participants were required to comply with standard testing protocols, including refraining from intense exercise in the preceding 12 h, and consuming very large meals or caffeinated products within the four hours preceding the scan [28]. A Lunar DPX pencil beam DEXA scanner (GE Healthcare, Madison, WI, USA) was used, and data were analysed using GE enCORE software (version 13.60; GE Healthcare) and a reference database. Protocols for positioning, analysis, and interpretation of results were undertaken in accordance with the Official Position of the International Society for Clinical Densitometry and compared to NHANES 1999–2004 reference data [29]. Data collected included fat-free mass (FFM), fat mass (FM), and bone mineral content/density (BMC/BMD). Android vs. gynoid fat mass distribution, as well as ratios between these indices, were also reported. Height-normalised indices of body composition were determined, including FFM index (FFMI) as FFM/height^2^ (kg/m^2^) and FM index (FMI) as FM/height^2^ (kg/m^2^) [30]. The participants were provided with feedback on the DEXA results (and counselling if required or requested) by an accredited dietitian.

### 2.2. Milk Sampling Protocol

A single human milk sample was collected following the third (and final) day of the dietary recall surveys, at 3–4 months postpartum. Participants were asked to obtain milk from each breast separately between 07:00 and 11:00 a.m., having not fed or expressed for at least two hours prior to sample collection [31]. Complete expressions from each breast were collected to ensure that a mixture of fore- and hind-milk was obtained. After mixing the expressed milk, 30 mL was transferred into a pre-labelled sterile container and stored in the participant’s refrigerator. The sample was collected within 24 h, and then the aliquots were labelled with codes and subsequently stored at −80 °C at the UniSC research laboratory until analysis [10,32].

### 2.3. Human Milk Compositional Analyses

The concentration of lactose in the human milk samples was determined using a spectrophotometric enzymatic assay as described by Mitoulas et al. (2002) [33]. The concentration of protein in the human milk sample was determined using a bicinchoninic acid (BCA) assay [34], and the human milk fat content was estimated using a modified creamatocrit method, based on the original validation work of Lucas et al. [35]. The metabolizable energy content of the milk samples (*n* = 101) was calculated using Atwater conversion factors [36,37].

The concentration of HMOs in the milk samples was quantitively determined using the Agilent UPLC (1290)/triple quadrupole MS (6470) at UniSC. Commercial-grade oligosaccharides (Glycom A/S) were used for the quantification of HMOs including neutral-core, fucosylated and acidic HMOs (2′FL, 2′-Fucosyllactose; 3FL, 3-Fucosyllactose; DFL, DiFucosyllactose; LNT, Lacto-*N*-tetrose; 3′SL, 3′-Sialyllactose; 6′SL, 6′-Sialyllactose; LNnT, Lacto-*N*-neotetraose; LNFP I/II/III, Lacto-*N*-fucopentaose-I/II/III; LSTa/b/c, Sialyl-lacto-*N*-tetraose a/b/c). The milk sample pre-treatment protocol was based on the method by Tonon et al. (2019) [38]. Frozen human milk samples were sonicated and brought to room temperature and vortexed, and then 300 µL was pipetted in duplicate into 500 µL microcentrifuge tubes (Thomas Scientific, Swedesboro, NJ, USA). The milk was centrifuged at 5000× *g*, 4 °C for 15 min, and the upper portion consisting predominantly of fat was removed. The defatted (skim) milk was diluted 20 times (20×) with ultrapure water (50 µL milk, 950 µL ultrapure water, Chromatography Grade, LiChrosolv 1153332500, Supelco, MilliporeSigma, Burlington, MA, USA), and transferred to Amicon 10 kDa molecular cut-off filters (0.5 mL) (Merck, Rahway, NJ, USA). The filters were centrifuged at 12,000× *g*, 4 °C for 30 min to remove proteins, and the resultant filtrate was retained for HMO analysis. Because of the range of HMO concentrations in human milk, two further dilutions of the milk filtrates were prepared at 60 times (60×), and 600 times (600×) and transferred to glass vials for LC-MS analysis. HMOs were separated using a Waters amide column (1.7 μm, 2.1 × 100 mm) which was equilibrated with Solution A (10 mmol/L ammonium formate, pH 7.2, Sigma-Aldrich, St. Louis, MO, USA) at a temperature of 50 °C and a rate of flow of 0.3 mL/min. The injection volume of each sample was 1.0 μL. The optimised gradient corresponding to Solution A and relative to Solution B (acetonitrile, Chromatography Grade, LiChrosolv 1000292500) involved the following: 5–75% (0–10 min), 75% (10–15 min), 75–65% (15–20 min), 65–10% (20–21 min), 10% (21–24 min), 10–5% (24–25 min), and 5% (25–35 min) [39]. HMOs were subsequently identified using an Agilent triple quadrupole MS using the multi-reaction monitoring (MRM) setting, and data was analysed using Agilent Mass Hunter software. Characteristic fragment ions from FUT2-dependent HMOs (2′FL and LNFP I) were used for classification of mothers as either secretor-positive or -negative (so-called “secretors”, or “non-secretors”).

### 2.4. Statistical Analyses

All analyses were performed using R (version 4.1.2), a *p*-Value < 0.05 was considered significant. Means and standard deviations were used to summarize continuous variables. Counts and percentages were used to summarize categorical variables. Welch’s two-sample *t*-test was used to compare groups for continuous variables. The groups tended to have approximately 50 or more observations, generally providing sufficient normality. Multiple comparisons were corrected by using either Bonferonni’s Method or the Benjamini–Hochberg method. Continuous variables were compared between three or more groups using the Kruskal–Wallis test. Dichotomous explanatory variables were formed by taking those observations at or below the median to be “low” and those above the median to be “high”.

## 3. Results

One hundred and one (*n* = 101) healthy, lactating women were recruited from the South-East Queensland community in Australia over a period of 12 months in 2021/2. All mothers completed Phase 1 and a subgroup of mothers who volunteered and consented (*n* = 30) completed Phase 2.

All participants birthed a term baby (mean gestational age 39.4 ± 1.4 weeks). The majority were Caucasian (93%), and most infants (83%) were exclusively breastfed until 3 months of age. The mean maternal pre-pregnancy BMI was 23.7 ± 3.6 kg/m^2^ (“normal” classification) [21]), and 47% met the criteria for appropriate rate of gestational weight gain (19% exceeded and 33% were below recommendations) [40]. There were reports of restricted or alternative dietary patterns amongst the participants (9% vegan/vegetarian, 2% gluten-free), and almost a quarter self-reported food allergies or intolerances. More than half (55%) of the participants used dietary supplements, most notably multi-vitamins and minerals (40%), as well as additional iron (14%) and omega 3 fatty acid (12%) supplements. Sixty percent of participants consumed caffeine, 3% reported alcohol use and none reported cigarette smoking. Further details are reported in Table 1.

### 3.1. Human Milk Composition

The mean macronutrient and energy composition of all human milk samples was within expected ranges for mature human milk and is presented in Table 2.

**Figure 2:**
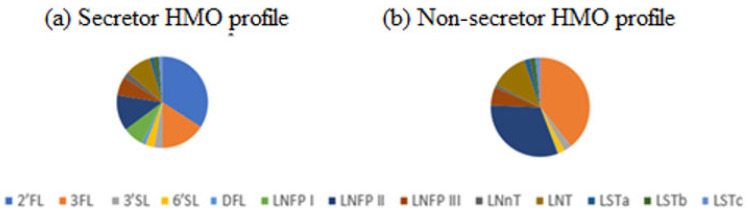
HMO profiles of secretor ((**a**): *n* = 80), and non-secretor ((**b**): *n* = 21) human milk samples from healthy Australian women (*n* = 101) at 3–4 months postpartum.

Thirteen major HMOs were quantified using LC-MS analysis in all human milk samples (*n* = 101) and the majority (79%) of the mothers were classified as “secretors”. As expected, levels of 2′FL, DFL, and LNFP I were significantly higher in secretor mothers’ milk samples, whilst neutral core HMOs were relatively higher in non-secretor milk. Table 3 presents the means and standard deviations of the HMOs overall and by secretor status, and Figure 2 illustrates the variation in HMO profiles by secretor status (refer to Table 3).

### 3.2. Associations between Human Milk Composition and Maternal Variables

There were no associations between human milk macronutrient and HMO composition, and maternal demographic variables (*p* > 0.05). There was no influence of the infant sex, nor method of delivery (vaginal vs. C-section). Lactose was significantly higher in mothers with only one child (parity = 1, *p =* 0.035), or in those that had gestation periods of >40 weeks (*p* = 0.006). Both fat and energy content of the breast milk were significantly higher when infants were born at a lower birthweight than the mean of 3.5 kg (*p =* 0.032 for both). LSTa was also higher in this case (*p =* 0.024). Total fat content was significantly lower (*p* = 0.039) and 6′SL significantly higher (*p =* 0.022) in milk sampled from mothers who exclusively breastfed their infants. Singular HMOs were associated with lactation stage: 2′FL, 6′SL, and DFL levels were higher in milk samples taken before 14 weeks postpartum (*p* < 0.05 for all). LSTa was higher in Caucasian mothers than those from other ethnic backgrounds (Asian, Hispanic, indigenous, *p =* 0.033). Maternal exclusion of food groups or food intolerances were not associated with any changes in human milk macronutrient or HMO composition. There were no significant effects of caffeine consumption, or maternal postpartum physical activity levels on HMO composition. LNT levels were negatively associated with maternal antibiotic exposure during pregnancy (*p =* 0.039). Maternal history of medical conditions such as thyroid dysfunction and allergy/atopy negatively impacted the concentrations of several HMOs, particularly LNT and several sialylated HMOs (6′SL, LSTa, LSTb, and LSTc, all *p* < 0.05). All findings were non-significant after adjusting for multiple comparisons, and individual observed associations would need to be confirmed in further studies with pre-planned hypotheses and singular comparisons.

### 3.3. Associations between HMO Profiles and Maternal Dietary Intake

**Figure 3:**
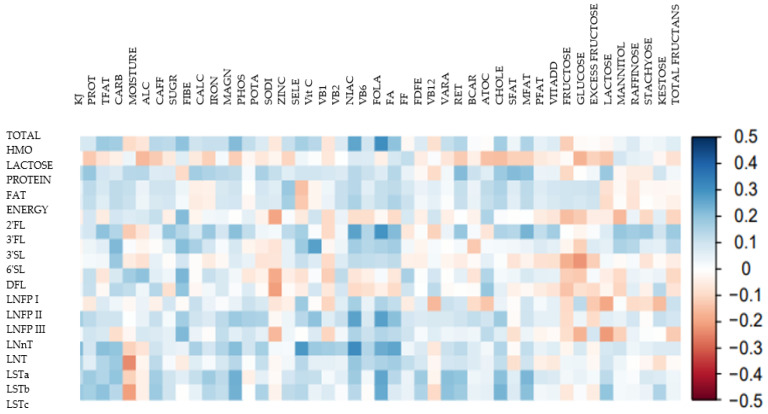
Correlation heatmap between milk components and average diet components, using Spearman’s correlation (*p* < 0.05). *p*-values have been adjusted for multiple correlations using the Benjamini–Hochberg method. Shading and colour reflect direction and strength of Spearman rank correlation coefficients (blue = negative; red = positive; white = no correlation; darker colour = stronger correlation).

There was no significant difference between the average nutrient composition of maternal diets over 3 days and nutrient composition of maternal diets in the 24 h immediately preceding milk sampling (*n* = 101, *p* > 0.9). Only those associations between nutrients and HMOs that were found to be significant on initial analysis are summarized in Table 4. There were no significant effects of dietary energy intake, total sugars, fibre, and total fat on any milk components. The only short-chain carbohydrate with an influence was stachyose (weakly correlated with LNFP I). There was no influence of fructose, mannitol, raffinose, or total fructans. Any level of alcohol consumption was associated with decreased levels of LSTa/b/c. Upon initial analysis, there were some associations between individual HMOs and selected minerals such as calcium, iron, and zinc, as well as the electrolytes sodium and potassium. Levels of vitamin B2 intake above the median intake for this group was associated with increased 3′SL levels, and vitamin B6 with higher LNFP III and LNT levels. Sialylated HMOs were moderately correlated with vitamin A, beta-carotene and alpha-tocopherol levels. Dietary folate (total, natural and folic acid) was initially strongly associated with the total HMO levels in the milk samples. All findings were non-significant after adjusting for multiple comparisons, as indicated by the correlation heatmap (Figure 3). Table 4 indicates raw *p*-Values, but, after applying an adjusted cut-off for all comparisons (a total of 3876) if the Bonferroni method was applied, this gives the very small adjusted cut-off of *p* = 0.000013, rendering no significant associations. Individual associations would need to be confirmed in further studies with singular hypotheses and fewer comparative variables. This study aimed to investigate a multi-nutrient diet and included many comparisons that reflect the complexity of studying free-living participants consuming varied diets.

### 3.4. Associations between HMO Profiles and Maternal Anthropometry

No associations were found between human milk macronutrient composition and HMO concentrations and maternal usual body weight, or pre-pregnancy BMI. Human milk protein was higher (10.69 g/L compared to 10.05 g/L, *p =* 0.04) when a mother’s delivery body weight was lower than the median (80 kg). LNnT was negatively associated with overall gestational weight gain above the median of 14 kg (*p =* 0.034), and with the rate of gestational weight gain (*p =* 0.022, median 0.36 kg/week).

### 3.5. Comparison of Maternal Body Composition Variables and Human Milk Values

DEXA results obtained from the maternal body composition analysis (*n* = 30) revealed that participants with a high fat-free mass index (FFMI) had human milk fat and energy contents that were significantly lower (*p =* 0.004 for both) than mean levels. Mothers with a high android fat mass as a percentage of total fat mass (% AFM), had significantly lower levels of LNFP III in their milk (374 ± 252 mg/L compared to 188 ± 166 mg/L, *p =* 0.025). However, these associations were not significant after correction for multiple comparisons. No significant associations were found between indices of fat mass or fat mass distribution values (presented in Table 5), and maternal height, and the composition of HMOs and human milk macronutrients in this cohort.

## 4. Discussion

The aim of this study was to examine macronutrient and HMO profiles in the mature human milk from Australian mothers, and to investigate any associations with maternal nutrition during lactation. The participants reflected a generally healthy cohort of lactating women with adequate dietary intake according to country-specific recommendations [44]. The proportion of mothers with positive secretor status and overall HMO concentrations in the milk samples were consistent with values reported for other, mainly Caucasian, populations [45].

In keeping with the literature, we found no major, consistent, and convincing influence of maternal dietary intake and body composition on HMO profiles. Maternal dietary intake has only been directly assessed in a limited number of observational studies [8,13,15,16,17]. Two of these studies were based on data from food frequency questionnaires (FFQ) to retrospectively assess the usual frequency of consumption of foods and beverages during pregnancy, not during lactation. Results from previous studies indicated that dietary fibre, polyphenols, and MUFAs had some effects on HMOs, and weak negative correlations were also noted for protein and empty calories [8,13]. We also found some weak positive associations between MUFA and singular HMOs (LNFP III, LST b/c), as well as between dietary protein and LNFP III and LSTc (all *p* < 0.05 before multiple comparison correction), but none for dietary fibre. Quin et al. [15] used a single dietary recall in the postpartum period, in a sample statistically too small to assess diet quality (*n* = 16). They did, however, find significant correlations between some dietary components (total sugars, dietary fibre) and the fucose/galactose in HMOs (*p* < 0.05). They suggested that maternal diet may influence the biosynthesis of HMOs, since the biosynthetic pathway initiates from activated monosaccharides. This is similar to the conclusion that may be drawn from a cross-over intervention trial (feeding study) which evaluated drastic manipulations of the usual diet in a controlled, inpatient setting (*n* = 7) [14]. Here, a high-fat diet (contributing > 40% total energy) resulted in a decrease in the concentration of sialylated HMOs as compared with a high-carbohydrate diet, and a higher-glucose versus higher-galactose diet affected the profiles of fucosylated HMOs. Since the participants’ dietary intake was unnaturally and drastically altered, this study has limited generalisability to the usual dietary intake of women during lactation, and it would be challenging to formulate practical dietary advice to lactating women from the findings.

In terms of dietary micronutrient intake, we report some associations between individual nutrients and HMOs upon initial analyses, but these were not significant after correcting for multiple comparisons. Two observational studies based in China used FFQs to assess maternal diet during lactation [16,17]. Qiao et al. [17], found that higher dietary vitamin A intakes (from food sources: 602.22 ± 126.46 µg/day) were associated with higher concentrations of sialic acid in breast milk (*p =* 0.000), with sialic acid being a component of sialylated HMOs. Similarly, we noted an initial significant association between dietary vitamin A and higher levels of sialylated HMOs, LSTa, and LSTc (both *p* < 0.05). Li et al. [16] repeated FFQs over time to provide insights into the longitudinal changes in HMO profiles over the course of lactation. They reported significant, positive associations between selected micronutrients and a few individual HMOs, specifically, vitamins A, C, B1, and B2 with fucosylated HMOs, and tocopherol with 3′SL [16]. We also noted initial weak, and somewhat different and therefore inconsistent, correlations between B-group vitamins and core or fucosylated HMOs, and alpha-tocopherol with sialylated HMOs.

The most notable finding from this study was the trend towards the trend towards positive associations between all forms of folate and total folate, and the concentration of total HMOs and some individual HMOs, as well as some individual HMOs. Dietary folate equivalent (DFE) was used to denote both natural food folate and fortification, and the mean maternal intake of 600 μg/day (*n* = 101) was above the RDI (500 μg/day) for lactating women. Intakes above this mean, and therefore the RDI, were associated with higher total HMO concentrations in the milk samples (*p* = 0.033, not significant after correction for multiple comparisons). The implication of this finding and the mechanism driving the association requires further investigation. It is known that folate secretion into the milk is not generally affected by maternal folate status or supplementation after reaching a threshold, if the mother has adequate folate status [46]. There may, however, be some merit in ensuring adequate folate status in clinically deficient mothers, or, at the very least, encouraging plant-based and fortified foods rich in folate in the usual diet when breastfeeding. Australian food databases list cereal products, vegetables, legumes, and fruit—in particular, fortified orange juices—as major dietary sources of folate [47]. Evidence suggests that there is a decrease in diet quality (along with fruit and vegetable intake) in the postpartum period, and addressing diet quality would nevertheless be beneficial for maternal nutritional health overall [48].

Previous studies have assessed maternal anthropometric characteristics as indicators of nutritional status besides dietary intake, including BMI. It has been proposed that glycosylation (and therefore HMO composition) may be influenced by maternal physiological status. Results have generally been conflicting: there is some evidence of correlations between maternal pre-pregnancy BMI and individual HMOs [9], yet the opposite has also been reported [10]. Postpartum measures of maternal BMI have been reported to positively correlate with 2′FL and other fucosylated HMOs [18,20], and inversely with others such as DSLNT and LNnT [7]. In interpreting these differences, it must be acknowledged that BMI is a crude measure of body composition, and generally not a good indicator of adiposity [49]. To address the potential association between maternal body composition and HMO profiles more thoroughly, a gold-standard technique (DEXA) and direct quantitation of HMOs were undertaken in this study. No significant associations between HMO concentrations and any of the body composition measurements and indices were found in the subgroup of thirty lactating women. It may be that the prevailing influence of genetic variation in lactating mothers overshadows any impact of maternal nutritional status, which requires further investigation.

This study is the first to explore associations between short-term maternal dietary intake and nutritional status during lactation, and the composition of quantitated HMOs in mature human milk samples. Limitations include the use of self-reported dietary data, limited generalisability of the findings to other populations, the use of only a few HMO standards, and the cross-sectional nature of the observations. Despite these limitations, this study is strengthened by its use of gold-standard nutrition and body-composition-assessment tools, and HMO analysis by LC-MS.

Future lactation research should also calculate the absolute amount of milk (and therefore HMOs) ingested by the infant, and relate findings to infant health and developmental outcomes, as a more holistic approach. Research into the influence of maternal nutrition on HMO concentrations in malnourished or diseased populations may be warranted, and other methods for assessing dietary quality, such as dietary pattern analyses, could be used to further explore relationships between diet and HMOs. Investigations into colostrum, transitional or pasteurised donor human milk, and longitudinal assessments across lactation would be of interest.

## 5. Conclusions

It has been hypothesised that maternal nutrition may affect HMO concentrations, perhaps by influencing the activity of the glycosyltransferases, or ensuring the availability of substates for HMO biosynthesis. The findings from this comprehensive investigation argue that the evidence for significant and consistent associations between maternal dietary exposures in healthy, general populations and HMO concentrations remains conflicting or unconvincing. It may be that moderate variations in maternal nutritional status, or short-term suboptimal dietary intake of nutrients in otherwise healthy mothers, does not have notable effects on HMO concentrations, as is the case for gross human milk macronutrient composition [50]. However, the link between maternal nutrition and HMO composition in cases of suboptimal nutritional and health status should be explored.

## Figures and Tables

**Table 1:** Selected characteristics of the study population (*n* = 101). Results are presented as a percentage (%) of the population for categorical variables and as a mean ± SD for continuous variables.

Variable and Category	Results
Age:	
20–30 years	35%
31–40 years	64%
40+ years	1%
Ethnicity:	
Caucasian	93%
Indigenous	1%
Other	6%
Parity:	
1	42%
2	44%
3+	14%
Time postpartum (weeks)	14.4 ± 4.4
Gestation period (weeks)	39.4 ± 1.4
Sex of baby:	
Male	67%
Female	33%
Birthweight of baby (kg)	3.5 ± 0.4
Usual body weight (pre-pregnancy) (kg)	66.3 ± 10.3
Body weight at time of delivery (kg)	80.7 ± 11.0
Pre-pregnancy BMI:	
Underweight	4%
Normal	68%
Overweight	24%
Obese	4%
Gestational weight gain (total, kg):	
Meets IOM guidelines	43%
Exceeded IOM guidelines	40%
Below IOM guidelines	17%
Current BMI at time of sampling:	
Underweight	2%
Normal	45%
Overweight	45%
Obese	8%

(BMI, body mass index as kg/m^2^; SD, standard deviation; IOM, Institute of Medicine).

**Table 2:** Human milk sample gross compositional data for all women compared with reference values (*n* = 101).

Milk Component	Mean ± SD	Reference Range [41,42,43]
Lactose (g/L)	75.4 ± 4.1	67–78
Protein (g/L)	10.4 ± 1.5	8–10
Fat (g/L)	39.3 ± 12.6	35–40
Energy (kcal/L)	697.3 ± 111.8	650–700

**Table 3:** HMO concentrations in mature human milk samples from a cohort of healthy Australian women (*n* = 101, at 3–4 months postpartum). Values are presented as mean ± SD.

HMO	Mean Concentration (mg/L) All Mothers (*n =* 101)	Mean Concentration (mg/L) Secretor Mothers (*n* = 80)	Mean Concentration (mg/L) Non-Secretor Mothers (*n* = 21)	*p*-Value ^1^
2′FL	1133 ± 982	1431 ± 889	1 ± 5	<0.001 *
3FL	937 ± 937	665 ± 573	1970 ± 1297	<0.001 *
3′SL	126 ± 87	127 ± 88	125 ± 87	>0.9
6′SL	127 ± 93	132 ± 97	105 ± 77	0.2
DFL	49 ± 57	59 ± 56	11 ± 45	<0.001 *
LNFP I	245 ± 267	308 ± 266	4 ± 11	<0.001 *
LNFP II	747 ± 823	530 ± 429	1571 ± 1327	0.002 *
LNFP III	284 ± 204	281± 189	296 ± 259	0.8
LNnT	79 ± 108	88 ± 110	46 ± 94	0.088
LNT	438 ± 303	392 ± 272	614 ± 355	0.013 *
LSTa	68 ± 60	61 ± 49	94 ± 88	0.12
LSTb	73 ± 70	68 ± 61	91 ± 97	0.3
LSTc	61 ± 59	56 ± 55	80 ± 70	0.14
TOTAL HMO	4365 ± 2142	4196 ± 1978	5009 ± 2632	0.2
Total Fucosylated	3394 ± 1723	3273 ± 1586	3854 ± 2152	0.3
Total Sialylated	454 ± 279	444 ± 256	495 ± 357	0.5
Total Neutral Core	517 ± 330	479 ± 314	660 ± 361	0.045 *

^1^ Welch two-sample *t*-test. * denotes significance.

**Table 4:** Associations between short-term maternal dietary intake during lactation and HMO concentrations in mature human milk samples from a cohort of healthy Australian women (*n* = 101), found to be significant on initial analysis, prior to correction for multiple comparisons. Associations between variables were calculated using correlation analyses.

Nutrient	Median	HMO	HMO Concentrations (mg/L) at Low Intake (Below Median)	HMO Concentrations (mg/L) at High Intake (Above Median)	*p*-Value
Protein	97 g/day	LNFP III	244 ± 137	325 ± 250	0.048
		LSTa	55 ± 33	82 ± 77	0.024
		LSTc	43 ± 28	78 ± 75	0.003
Carbohydrate	236 g/day	DFL	61 ± 70	36 ± 36	0.024
Alcohol	0 g/day	LSTa	75 ± 64	46 ± 38	0.008
		LSTb	78 ± 77	54 ± 38	0.043
		LSTc	67 ± 63	41 ± 37	0.015
Calcium	929 mg/day	3′SL	105 ± 70	148 ± 97	0.013
		LNnT	53 ± 87	105 ± 121	0.014
Iron	13 mg/day	3′SL	102 ± 67	151 ± 98	0.004
		LNFP III	241 ± 148	327 ± 243	0.035
Potassium	3328 mg/day	3FL	749 ± 768	1128 ± 1057	0.042
		LNFP III	235 ± 131	333 ± 250	0.016
		LNT	364 ± 244	513 ± 340	0.013
		LSTb	58 ± 42	87 ± 88	0.036
		LSTc	47 ± 41	74 ± 70	0.021
Sodium	2517 mg/day	LNFP III	242 ± 142	327 ± 247	0.038
		2′FL	931 ± 908	1339 ± 1020	0.036
Zinc	12 mg/day	LNFP III	236 ± 143	333 ± 244	0.017
Selenium	92 mg/day	2′FL	1342 ± 1001	920 ± 924	0.03
Vit B2	2 mg/day	3′SL	108 ± 69	145 ± 99	0.033
Vit B6	1.5 mg/day	LNFP III	242 ± 148	327 ± 243	0.038
		LNT	378 ± 295	499 ± 302	0.044
Folate (total) ^1^	506 µg/day	Total HMO	3802 ± 1951	4940 ± 2193	0.007
		3FL	722 ± 710	1155 ± 1087	0.02
		LNFP II	565 ± 500	932 ± 1028	0.026
		LNFP III	232 ± 170	337 ± 224	0.009
		LNT	337 ± 269	541 ± 304	<0.001
Folate, DFE	600 µg/day	Total HMO	3918 ± 2017	4821 ± 2188	0.033
Folate, natural	341 µg/day	Total HMO	3627 ± 1621	5118 ± 2352	<0.001
		3FL	676 ± 672	1202 ± 1090	0.005
		LNFP II	574 ± 527	923 ± 1018	0.034
		LNFP III	225 ± 126	343 ± 248	0.004
		LNT	342 ± 218	536 ± 346	0.001
		LSTa	53 ± 34	83 ± 76	0.012
		LSTb	57 ± 45	89 ± 86	0.023
		LSTc	47 ± 41	74 ± 70	0.021
RAE ^2^	945 µg/day	LSTa	55 ± 30	81 ± 78	0.031
		LSTc	48 ± 39	73 ± 72	0.035
Beta-Carotene	2932 µg/day	LSTa	54 ± 30	83 ± 78	0.018
		LSTb	59 ± 43	87 ± 88	0.047
A-tocopherol ^3^	13 mg/day	LSTb	58 ± 45	87 ± 87	0.038
		LSTc	47 ± 39	74 ± 71	0.022
MUFA	40 mg/day	LNFP III	44 ± 136	325 ± 251	0.047
		LSTb	58 ± 42	88 ± 88	0.035
		LSTc	46 ± 34	75 ± 73	0.013
Stachyose	0.3 g/day	LNFP I	190 ± 234	300 ± 289	0.038

Notes: Vitamin D (D2 + D3) (µg) and Vitamin K, phylloquinone (µg) are not available in the ASA24-Australia-2016^®^ version. Database used: ASA24-Australia-2016: Australian Food, Supplement and Nutrient Database (AUSNUT) 2011–2013 Nutrient File *. ^1^ Folate, Total: natural folate + folic acid. Folate, Natural: folate values without added folic acid. Folate, DFE: dietary folate equivalent, natural folate + (folic acid * 1.67). ^2^ RAE (retinol activity equivalent) vitamin A: retinol + (beta-carotene/6) + (alpha-carotene/12) + (cryptoxanthin/12). ^3^ A-tocopherol: vitamin E, alpha-tocopherol is used in the calculation of vitamin E by FSANZ.

**Table 5:** Body composition indices of healthy, lactating Australian women using DEXA scans (*n* = 30). Results are presented as mean ± SD and range.

	FM (kg)	FM (%)	FMI (kg/m^2^)	FFM (g)	FFMI (kg/m^2^)	FM:FFM Proportion	AFM:GFM Proportion
Mean ± SD	25.4 ± 8.2	36 ± 7	9.6 ± 3.1	42.7 ± 4.6	17.4 ± 1.3	0.6 ± 0.2	0.4 ± 0.1
Range	10.9–44.5	20–50	4–17	34.7–54.0	5.2–18.7	0.2–0.9	0.2–0.5

Notes: FM, total fat mass; % FM, % fat mass of total body mass; FMI, fat mass index as FM/Height^2^; LBM, total lean body mass; FFM, total fat-free mass; FFMI, fat-free mass index as FFM/Height^2^; FM/FFM, ratio of FM:FFM; AFM:GFM, proportion android:gynoid FM.

## Data Availability

The data presented in this study are available on request from the corresponding author. The data are not publicly available due to thesis examination process currently underway.

## References

[1] Ballard O., Morrow A.L. (2013). Human milk composition: Nutrients and bioactive factors. Pediatr. Clin. N. Am..

[2] Bode L. (2012). Human milk oligosaccharides: Every baby needs a sugar mama. Glycobiology.

[3] Kunz C., Rudloff S. (2017). Compositional analysis and metabolism of human milk oligosaccharides in infants. Nestlé Nutr. Inst. Workshop Ser..

[4] Jantscher-Krenn E., Bode L. (2012). Human milk oligosaccharides and their potential benefits for the breast-fed neonate. Minerva Pediatr..

[5] Thurl S., Munzert M., Henker J., Boehm G., Müller-Werner B., Jelinek J., Stahl B. (2010). Variation of human milk oligosaccharides in relation to milk groups and lactational periods. Br. J. Nutr..

[6] Coppa G.V., Gabrielli O., Pierani P., Catassi C., Carlucci A., Giorgi P.L. (1993). Changes in carbohydrate composition in human milk over 4 months of lactation. Pediatrics.

[7] McGuire M.K., Meehan C.L., McGuire M.A., Williams J.E., Foster J., Sellen D.W., Kamau-Mbuthia E.W., Kamundia E.W., Mbugua S., Moore S.E. (2017). What’s normal? Oligosaccharide concentrations and profiles in milk produced by healthy women vary geographically. Am. J. Clin. Nutr..

[8] Azad M.B., Robertson B., Atakora F., Becker A.B., Subbarao P., Moraes T.J., Mandhane P.J., Turvey S.E., Lefebvre D.L., Sears M.R. (2018). Human Milk Oligosaccharide Concentrations Are Associated with Multiple Fixed and Modifiable Maternal Characteristics, Environmental Factors, and Feeding Practices. J. Nutr..

[9] Ferreira A.L., Alves R., Figueiredo A., Alves-Santos N., Freitas-Costa N., Batalha M., Yonemitsu C., Manivong N., Furst A., Bode L. (2020). Human Milk Oligosaccharide Profile Variation Throughout Postpartum in Healthy Women in a Brazilian Cohort. Nutrients.

[10] Samuel T.M., Binia A., de Castro C.A., Thakkar S.K., Billeaud C., Agosti M., Al-Jashi I., Costeire M.J., Marchinr G., Martinez-Costa C. (2019). Impact of maternal characteristics on human milk oligosaccharide composition over the first 4 months of lactation in a cohort of healthy European mothers. Sci. Rep..

[11] Davis J.C., Lewis Z.T., Krishnan S., Bernstein R.M., Moore S.E., Prentice A.M., Mills D.A., Lebrilla C.B., Zivkovic A.M. (2017). Growth and Morbidity of Gambian Infants are Influenced by Maternal Milk Oligosaccharides and Infant Gut Microbiota. Sci. Rep..

[12] Biddulph C., Holmes M., Kuballa A., Davies P.S.W., Koorts P., Carter R.J., Maher J. (2021). Human Milk Oligosaccharide Profiles and Associations with Maternal Nutritional Factors: A Scoping Review. Nutrients.

[13] Selma-Royo M., González S., Gueimonde M., Chang M., Fürst A., Martínez-Costa C., Bode L., Collado M.C. (2022). Maternal Diet Is Associated with Human Milk Oligosaccharide Profile. Mol. Nutr. Food Res..

[14] Seferovic M.D., Mohammad M., Pace R.M., Engevik M., Versalovic J., Bode L., Haymond M., Aagaard K.M. (2020). Maternal diet alters human milk oligosaccharide composition with implications for the milk metagenome. Sci. Rep..

[15] Quin C., Vicaretti S.D., Mohtarudin N.A., Garner A.M., Vollman D.M., Gibson D.L., Zandberg W.F. (2020). Influence of sulfonated and diet-derived human milk oligosaccharides on the infant microbiome and immune markers. J. Biol. Chem..

[16] Li X., Mao Y., Liu S., Wang J., Li X., Zhao Y., Hill D.R., Wang S. (2022). Vitamins, Vegetables and Metal Elements Are Positively Associated with Breast Milk Oligosaccharide Composition among Mothers in Tianjin, China. Nutrients.

[17] Qiao Y., Feng J., Yang J., Gu G. (2013). The relationship between dietary vitamin A intake and the levels of sialic acid in the breast milk of lactating women. J. Nutr. Sci. Vitaminol..

[18] Isganaitis E., Venditti S., Matthews T.J., Lerin C., Demerath E.W., Fields D.A. (2019). Maternal obesity and the human milk metabolome: Associations with infant body composition and postnatal weight gain. Am. J. Clin. Nutr..

[19] Larsson M.W., Lind M.V., Laursen R.P., Yonemitsu C., Larnkjær A., Mølgaard C., Michaelsen K.F., Bode L. (2019). Human Milk Oligosaccharide Composition Is Associated with Excessive Weight Gain during Exclusive Breastfeeding—An Explorative Study. Front. Pediatr..

[20] Tonon K.M., de Morais M.B., Abrão A.C.F.V., Miranda A., Morais T.B. (2019). Maternal and Infant Factors Associated with Human Milk Oligosaccharides Concentrations According to Secretor and Lewis Phenotypes. Nutrients.

[21] Garrow J.S., Webster J. (1985). Quetelet’s index (W/H2) as a measure of fatness. Int. J. Obes..

[22] Schlaff R.A., Baruth M., Deere S.J., Boggs A., Odabasic A. (2020). Associations between prenatal diet quality and gestational weight gain. Nutr. Health.

[23] Widaman A.M., Keim N.L., Burnett D.J., Miller B., Witbracht M.G., Widaman K.F., Laugero K.D. (2017). A Potential Tool for Clinicians; Evaluating a Computer-Led Dietary Assessment Method in Overweight and Obese Women during Weight Loss. Nutrients.

[24] Rhee J.J., Sampson L., Cho E., Hughes M.D., Hu F.B., Willett W.C. (2015). Comparison of methods to account for implausible reporting of energy intake in epidemiologic studies. Am. J. Epidemiol..

[25] Biesiekierski J.R., Rosella O., Rose R., Liels K., Barrett J.S., Shepherd S.J., Gibson P.R., Muir J.G. (2011). Quantification of fructans, galacto-oligosacharides and other short-chain carbohydrates in processed grains and cereals. J. Hum. Nutr. Diet..

[26] Muir J.G., Rose R., Rosella O., Liels K., Barrett J.S., Shepherd S.J., Gibson P.R. (2009). Measurement of short-chain carbohydrates in common Australian vegetables and fruits by high-performance liquid chromatography (HPLC). J. Agric. Food Chem..

[27] Muir J.G., Shepherd S.J., Rosella O., Rose R., Barrett J.S., Gibson P.R. (2007). Fructan and free fructose content of common Australian vegetables and fruit. J. Agric. Food Chem..

[28] Heyward V.H., Wagner D.R. (2004). Applied Body Composition Assessment.

[29] Shepherd J.A., Ng B.K., Sommer M.J., Heymsfield S.B. (2017). Body composition by DXA. Bone.

[30] Gridneva Z., Rea A., Tie W.J., Lai C.T., Kugananthan S., Ward L.C., Murray K., Hartmann P.E., Geddes D.T. (2019). Carbohydrates in Human Milk and Body Composition of Term Infants during the First 12 Months of Lactation. Nutrients.

[31] Williams J.E., Price W.J., Shafii B., Yahvah K.M., Bode L., McGuire M.A., McGuire M.K. (2017). Relationships among Microbial Communities, Maternal Cells, Oligosaccharides, and Macronutrients in Human Milk. J. Hum. Lact..

[32] Wang J., Johnson T., Sahin L., Tassinari M.S., Anderson P.O., Baker T.E., Bucci-Rechtweg C., Burckart G.J., Chambers C.D., Hale T.W. (2017). Evaluation of the Safety of Drugs and Biological Products Used during Lactation: Workshop Summary. Clin. Pharmacol. Ther..

[33] Mitoulas L.R., Kent J.C., Cox D.B., Owens R.A., Sherriff J.L., Hartmann P.E. (2002). Variation in fat, lactose and protein in human milk over 24 h and throughout the first year of lactation. Br. J. Nutr..

[34] Keller R.P., Neville M.C. (1986). Determination of total protein in human milk: Comparison of methods. Clin. Chem..

[35] Lucas A., Gibbs J.A., Lyster R.L., Baum J.D. (1978). Creamatocrit: Simple clinical technique for estimating fat concentration and energy value of human milk. Br. Med. J..

[36] Perrin M.T., Belfort M.B., Hagadorn J.I., McGrath J.M., Taylor S.N., Tosi L.M., Brownell E.A. (2020). The Nutritional Composition and Energy Content of Donor Human Milk: A Systematic Review. Adv. Nutr..

[37] Fenton T.R., Elmrayed S. (2021). The Importance of Reporting Energy Values of Human Milk as Metabolizable Energy. Front. Nutr..

[38] Tonon K.M., Miranda A., Abrão A., de Morais M.B., Morais T.B. (2019). Validation and application of a method for the simultaneous absolute quantification of 16 neutral and acidic human milk oligosaccharides by graphitized carbon liquid chromatography-electrospray ionization-mass spectrometry. Food Chem..

[39] Zhang W., Wang T., Chen X., Pang X., Zhang S., Obaroakpo J.U., Shilong J., Lu J., Lv J. (2019). Absolute quantification of twelve oligosaccharides in human milk using a targeted mass spectrometry-based approach. Carbohydr. Polym..

[40] Rasmussen K.M., Yaktine A.L. (2009). Weight Gain during Pregnancy: Reexamining the Guidelines.

[41] Kim S.Y., Yi D.Y. (2020). Components of human breast milk: From macronutrient to microbiome and microRNA. Clin. Exp. Pediatr..

[42] Picciano M.F. (2001). Nutrient composition of human milk. Pediatr. Clin. N. Am..

[43] Hale T.W., Hartmann P.E. (2007). Hale & Hartmann’s Textbook of Human Lactation.

[44] National Health and Medical Research Council (2006). Nutrient Reference Values for Australia and New Zealand. Commonwealth Department of Health and Ageing: Australia.

[45] Thum C., Wall C.R., Weiss G.A., Wang W., Szeto I.M.-Y., Day L. (2021). Changes in HMO Concentrations throughout Lactation: Influencing Factors, Health Effects and Opportunities. Nutrients.

[46] Allen L.H. (2012). B vitamins in breast milk: Relative importance of maternal status and intake, and effects on infant status and function. Adv. Nutr..

[47] Food Standards Australia New Zealand (2014). AUSNUT 2011–13–Australian Food Composition Database.

[48] Lee Y.Q., Loh J., Ang R.S.E., Chong M.F.-F. (2020). Tracking of Maternal Diet from Pregnancy to Postpregnancy: A Systematic Review of Observational Studies. Curr. Dev. Nutr..

[49] Nuttall F.Q. (2015). Body Mass Index: Obesity, BMI, and Health: A Critical Review. Nutr. Today.

[50] Bravi F., Wiens F., Decarli A., Dal Pont A., Agostoni C., Ferraroni M. (2016). Impact of maternal nutrition on breast-milk composition: A systematic review. Am. J. Clin. Nutr..

